# Varieties and ensiling: Impact on chemical composition, fermentation quality and bacterial community of alfalfa

**DOI:** 10.3389/fmicb.2022.1091491

**Published:** 2023-01-11

**Authors:** Jianyu Lin, Guanhua Li, Lin Sun, Shuang Wang, Xin Meng, Licong Sun, Lin Yuan, Linbo Xu

**Affiliations:** ^1^Inner Mongolia Engineering Technology Research Center of Germplasm Resources Conservation and Utilization, School of Life Sciences, Inner Mongolia University, Hohhot, China; ^2^Inner Mongolia Key Laboratory of Microbial Ecology of Silage, Inner Mongolia Engineering Research Center of Development and Utilization of Microbial Resources in Silage, Inner Mongolia Academy of Agriculture and Animal Husbandry Sciences, Hohhot, China; ^3^Key Laboratory of Biohazard Monitoring and Green Prevention and Control for Artificial Grassland, Ministry of Agriculture and Rural Affairs, Institute of Grassland Research, Chinese Academy of Agricultural Sciences, Hohhot, China

**Keywords:** alfalfa, silage, chemical composition, fermentation quality, bacterial community

## Abstract

**Introduction:**

Six species of alfalfa commonly found in northern China were collected in the present study.

**Methods:**

The chemical composition and epiphytic microbial communities during the ensiling were analyzed; and their effects on fermentation quality and silage bacterial communities were assessed. The effects of physicochemical characteristics of alfalfa on the bacterial community were also investigated in terms of nutritional sources of microbial growth and reproduction.

**Results and discussion:**

The results showed that the chemical composition was significantly different in various alfalfa varieties, yet, the dominant genera attached to each variety of alfalfa was similar, except for pantoea (*p*<0.05). After ensiling, both the fermentation quality and microbial community changed obviously (*p*<0.05). Specifically, ZM2 had lower pH and ammonia nitrogen (NH_3_-N) content but higher LA content than other varieties of alfalfa silage. Beneficial bacteria such as *Lentilactobacillus* and *Lactiplantibacillus* were predominant in ZM2, which accounted for the higher fermentation quality. Significant correlations between the chemical composition of silage, fermentation quality and bacterial communities composition were observed. Moreover, variations in bacteria community structure during the fermentation of alfalfa were mainly influenced by water-soluble carbohydrates (36.79%) and dry matter (21.77%).

**Conclusion:**

In conclusion, this study revealed the influence of chemical composition on microbial community and fermentation quality, laying the groundwork for future studies on high-quality silage.

## Introduction

Alfalfa (*Medicago sativa* L.) is a leguminous forage with high crude protein (CP) content and high feed value (Krakowska et al., 2017), which is widely grown worldwide for forage. In northern China or other countries and regions restricted by growing seasons, forage must be effectively preserved to feed animals. Therefore, feed maintenance has become an important aspect of ruminant feed. However, alfalfa hay processing is subject to many limitations, including substantial dry matter (DM) loss and microbial respiration even during rainfall (You et al., 2022). Silage is the process of lactic acid bacteria (LAB) fermentation of fresh forage feed under anaerobic conditions (Driehuis and Oude, 2000). In this respect, LAB can reportedly utilize the water-soluble carbohydrates (WSC) for growth and produce organic acids to lower the pH value and inhibit the growth of harmful microorganisms such as *Clostridium*, *Enterobacter* and mold, resulting in the reduction in CP and DM loss (Yang et al., 2020). Additionally, the organic acid produced during silage fermentation increases the appetite of animals, promotes animal feed intake, and increases their production performance (Na et al., 2022). Therefore, ensiling has become an effective method to preserve the nutritional value of alfalfa and improve animal performance.

Techniques used to enhance the quality of alfalfa fermentation include wilting treatments and the supplementation of LAB additives. The wilting treatment can inhibit the reproduction of harmful microorganisms and avoid the limitation of pH reduction and silage fermentation by higher buffer energy values, resulting in better silage fermentation quality (Zheng et al., 2018). The supplementation of LAB inoculant ensures that sufficient LAB is available to initiate fermentation in the pre-fermentation period, thus preventing undesirable microorganisms such as *Clostridium* from taking over. It is worth noting that wilting treatments and inoculation with general LAB inoculants do not improve the silage quality of alfalfa in some cases (Wu and Nishino, 2016; Zhao et al., 2020), which may be related to the epiphytic microorganisms of forages such as alfalfa and the WSC content and buffer energy value of forage varieties (Zhang et al., 2018; Zheng et al., 2018; Zhang et al., 2021). It is well-established that the fermentation quality of different varieties of forages can differ due to the heterogeneity in the physicochemical characteristics and epiphytic microbial communities of different forages. Therefore, it is necessary to study the chemical composition and epiphytic microbial communities of alfalfa to reveal the fermentation characteristics and bacterial community composition characteristics of different alfalfa varieties. The bacterial communities involved in silage fermentation warrant extensive investigation to reveal important taxa that could help improve the quality of alfalfa silage.

Herein, we collected six species of alfalfa commonly found in northern regions at the same time, place and growing period. The chemical composition and bacterial microbial community composition of these alfalfa species were analyzed. After ensiling, the fermentation quality and microbial community were studied. Overall, this study aimed to compare the effects of chemical composition and epiphytic microbial composition of different alfalfa varieties on silage fermentation quality and microbial composition to reveal the main factors affecting silage fermentation quality.

## Materials and methods

### Silage preparation

Different alfalfa varieties, including Zhongcao No. 3 (ZC), Zhongmu No. 1 (ZM1), Zhongmu No. 2 (ZM2), Gongnong No. 1 (GN), WL168 (WL) and Xinjiangdaye (XJD) were planted in the Agriculture and Animal Husbandry Interlaced Area Test Base of Institute of grassland research of caas in Shaerqin, Inner Mongolia Autonomous Region (111°45′E、40°34′N), China. Six varieties of squaring stage alfalfa were harvested on June 4, 2021, and wilted in the field for 12 h. The wilted forages from the field were chopped to 10–20 mm lengths using a hay cutter. The silage raw materials were mixed and packed into special vacuum packaging bags (food grade，250 mm × 350 mm, MAGIC SEAL, Guangdong, China) for silage; each bag was about 200 g, which was quickly vacuum sealed by a vacuum packaging machine (DZ-300; Qingye, Beijing, China). Every treatment was repeated three times, and all samples were stored for 60 days at room temperature (20–30°C).

### Fermentation quality and chemical composition

The chemical composition and fermentation quality of fresh and ensiled alfalfa were analyzed. DM content was determined by oven drying at 65°C for 48 h, according to Guo et al. (2018). The CP content was determined using a Kjeldahl nitrogen analyzer (Kjeflex K-360, BUCHI, Switzerland) (Patrica, 1997). The WSC content was determined by colorimetric after-reaction with anthrone reagent (Thomas, 1977). The neutral detergent fiber (NDF) and acid detergent fiber (ADF) contents were assessed using an Ankom A2000i fiber analyzer (A2000i, Ankom Technology, Macedon, NY, United States), according to the method described by Van Soest et al. (1991). Water extracts of silage were obtained by 10-fold dilution with distilled water, homogenizing through a sterile homogenizer (JX-05, Shanghai Jingxin Industrial Development Co., Ltd., Shanghai, China), and filtering through four layers of cheesecloth (Sun et al., 2021). The pH value of silage material and silage was recorded using a pH benchtop meter (METTLER TOLEDO; SevenExcellence, Switzerland). The lactic acid (LA), acetic acid (AA), propionic acid (PA) and butyric acid (BA) concentration was determined using HPLC (DAD, 210 nm, SPD-20A, Shimadzu Co., Ltd., Kyoto, Japan). NH_3_-N was determined using the phenol-hypochlorite reaction method, according to the method described by Broderick and Kang (1980).

### Bacterial diversity sequencing

The DNA extraction was performed according to the kit instructions (D4015, Omega Inc., Norcross, GA, United States), and the quality of the extracted DNA was detected by 1% agarose gel electrophoresis. The 16S rRNA gene (V3-V4 region) was amplified using the specific primers 799F (5′-AACMGGATTAGATACCCKG-3′) and 1193R (5′- ACGTCATCCCCACCTTCC-3′) with sample-specific barcodes. The bacterial 16S rDNA gene fragment was amplified by PCR (GeneAmp®9700; ABI, America). Each sample was replicated 3 times. The PCR products of the same sample were mixed and detected by 2% agarose gel electrophoresis. The AxyPrep DNA gel recovery kit (AXYGEN Company) was used to cut the gel to recover the PCR products, and Tris HCl was used for elution. Next, the PCR products were detected and quantified by QuantiFluor™-ST blue fluorescence quantitative system (Promega Company) and then mixed in the corresponding proportion according to the requirement of sequencing quantity of each sample. Finally, the PCR products were denatured with sodium hydroxide to generate single-stranded DNA fragments sequenced on the Illumina platform 250PE.

### Sequence data analysis

Trimmomatic software was used to filter the quality of reads and filter out reads below 50 bp. FLASH software was used to merge the paired reads into one sequence, and the minimum overlap length is 10 bp. Chimera removal was conducted using Usearch software and the Gold database. All sequences were divided into OTUs using Usearch software (Version 10) according to the similarity level of different sequences, and bioinformatics statistical analysis was usually performed on OTUs at the 97% similarity level. The species abundance index was calculated by Mothur software (Version 1.30.1) for sequences with an OTU similarity level of more than 97% (Schloss et al., 2011). PCA analysis at the bacterial genus level and species composition analysis at the bacterial phylum and genus levels were conducted using R (Version 3.2.1).

### Statistical analysis

The raw data were sorted by Excel 2010 software and statistically analyzed by SPSS 20.0 software. Data from different experimental groups were analyzed using one-way ANOVA and differences were compared using the least significant difference test. A value of *p* < 0.05 was statistically significant, and a value of *p* < 0.01 was highly statistically significant.

## Results

### Chemical composition of fresh alfalfa

The chemical composition of different varieties of alfalfa is shown in Table 1. Significant differences were found in the chemical composition of different alfalfa varieties (*p* < 0.05). The highest DM content was found in XJD and the lowest DM in ZC. The CP content of WL and the WSC content of XJD were significantly higher than the others (*p* < 0.05). The NDF content of alfalfa ranged from 364 to 419 g/kg DM, with ZM2 and WL having the highest and lowest NDF content, respectively. The ADF content of alfalfa ranged from 257 to 345 g/kg DM, with ZC and GN having the highest and lowest ADF content, respectively.

**Table 1:** Chemical composition of fresh alfalfa.

Items	ZC	ZM1	ZM2	GN	WL	XJD	SEM	*p*-value
DM (g/kg FM)	463.66d	464.33d	473.65d	497.22bc	506.51ab	513.53a	5.10	<0.001
CP (g/kg DM)	178.95bc	166.62c	167.76c	176.12bc	222.34a	187.93b	4.93	<0.001
WSC (g/kg DM)	38.37c	41.66c	44.64c	44.64c	57.73b	66.06a	2.48	<0.001
NDF (g/kg DM)	417.51ab	411.17abc	419.94a	382.35abcd	364.79d	387.98abcd	6.98	0.1
ADF (g/kg DM)	345.87a	308.08ab	287.59bc	257.24c	266.52c	267.7c	8.60	0.003

Values with different letters indicate significant differences between fresh materials. SEM, standard error of the mean; DM, dry matter; CP, crude protein; NDF, neutral detergent fiber; ADF, acid detergent fiber; WSC, water-soluble carbohydrate; ZC, Zhongcao No. 3; ZM1, Zhongmu No. 1; ZM2, Zhongmu No. 2; GN, Gongnong No. 1; WL, WL168; XJD, xinjiangdaye.

### Fermentation quality and chemical composition of alfalfa silage

The chemical composition of different alfalfa varieties after fermentation is shown in Table 2; Supplementary Table S1. One-way ANOVA showed that alfalfa silage fermentation significantly decreased the WSC content (*p* < 0.001). After 60 days of ensiling, the DM content of the silage ranged from 481 g/kg FM to 556 g/kg FM, with WL (556.15 g/kg FM) and XJD (545.02 g/kg FM) significantly higher than other alfalfa varieties (*p* < 0.05). The CP content of WL (204.23 g/kg DM) was significantly higher than the others (*p* < 0.05). Moreover, XJD exhibited the highest content of WSC, while ZC had the lowest WSC content. Finally, the NDF and ADF contents of XJD were significantly lower.

**Table 2:** Chemical composition of alfalfa silage after 60 days of ensiling.

Items	ZC	ZM1	ZM2	GN	WL	XJD	SEM	*P*-value
DM (g/kg FM)	481.64c	482.84c	481.72c	490.1c	556.15a	545.02ab	8.49	0.001
CP (g/kg DM)	183.17bc	185.29b	175.37 cd	171.86d	204.23a	172.75d	2.89	<0.001
WSC (g/kg DM)	13.4b	20.03ab	15.67b	22.22ab	21.4ab	23.38a	1.14	0.038
NDF (g/kg DM)	422.32a	387.52bcd	394b	393.79bc	374.21e	343.01f	5.95	<0.001
ADF (g/kg DM)	368.53a	305.31b	278.9bcd	269.31cde	281.26bc	245.92e	9.82	<0.001

Values with different letters indicate significant differences between silages. SEM, standard error of the mean; DM, dry matter; CP, crude protein; NDF, neutral detergent fiber; ADF, acid detergent fiber; WSC, water-soluble carbohydrate; ZC, Zhongcao No. 3; ZM1, Zhongmu No. 1; ZM2, Zhongmu No. 2; GN, Gongnong No. 1; WL, WL168; XJD, xinjiangdaye.

The fermentation quality of different varieties of alfalfa silage was described as shown in Table 3; Supplementary Table S1. One-way ANOVA showed that silage fermentation significantly affected the pH value and the content of NH_3_-N, LA, and AA. After 60 days of fermentation, the pH values of each type of alfalfa after ensiling ranged between 4.49 and 5.36, and the pH values of each variety of alfalfa after fermentation were significantly lower before fermentation (*p* < 0.05). There was a higher NH_3_-N content in WL and XJD, LA content in ZC, and AA content in XJD compared with other varieties of alfalfa silage (*p* < 0.05). The LA/AA values of ZC and ZM2 were significantly higher (*p* < 0.05). Furthermore, no PA or BA was detected in alfalfa silage from either species.

**Table 3:** Fermentation quality of alfalfa silage after 60 days of ensiling.

Items	ZC	ZM1	ZM2	GN	WL	XJD	SEM	*P*-value
PH	4.59e	4.86bc	4.49e	4.83bcd	4.9b	5.36a	0.31	<0.001
NH_3_-N (g/kg TN)	65.11c	48.35c	57.89c	55.31c	150.36a	132.6ab	10.15	<0.001
LA (g/kg DM)	54.02a	19.34c	42.27ab	24.43c	39.21 ac	36.35bc	3.28	0.005
AA (g/kg DM)	28.94ab	15.13d	21.32bcd	26.91abc	23.1abcd	33.46a	1.92	0.062
PA (g/kg DM)	ND	ND	ND	ND	ND	ND	–	–
BA (g/kg DM)	ND	ND	ND	ND	ND	ND	–	–
LA/AA	1.88ab	1.28abcd	2a	0.98d	1.76abc	1.13 cd	0.13	0.048

Values with different letters indicate significant differences between silages. SEM, standard error of the mean; DM, dry matter; NH_3_-N, ammonia nitrogen; LA, lactic acid; AA, acetic acid; PA, propionic acid; BA, butyric acid; ZC, Zhongcao No. 3; ZM1, Zhongmu No.1; ZM2, Zhongmu No.2; GN, Gongnong No.1; WL, WL168; XJD, xinjiangdaye.

### Bacterial community of alfalfa silage

High-throughput sequencing of the V3-V4 variable region of 16S rDNA of fresh alfalfa and alfalfa silage epiphytes was performed, and most bacteria were detected in all samples yielding a Good’s Coverage Index of approximately 1. The alpha diversity of bacterial communities was calculated and evaluated (Table 4). In this study, the Chao1, Shannon and ACE indices of epiphytic bacteria of different alfalfa varieties before ensiling were comparable (*p* = 0.268, *p* = 0.106, *p* = 0.223), indicating that each variety of alfalfa had the same bacterial composition before ensiling. After ensiling, the Chao1, Shannon, and ACE indices of alfalfa silage of each variety significantly decreased (*p* < 0.001, *p* < 0.001, *p* < 0.001), which indicated that the composition of alfalfa epiphytic bacterial community was influenced by fermentation during ensiling.

**Table 4:** Alpha diversity of the bacterial community in fresh materials and alfalfa silage.

Treatment	**Reads**	**Chao 1**	**Shannon**	**Ace**	**Coverage**
ZC0	45,647	1,802abc	5.34abc	1,776abc	0.99
ZM10	37,861	1,637de	5.38ab	1,642bcde	0.99
ZM20	36,503	1,903ab	5.60ab	1,873ab	0.99
GN0	47,241	2,066a	5.84a	2,021a	0.99
WL0	38,043	1,457def	4.72be	1,458ef	0.99
XJD0	45,296	1,729bcd	5.17abd	1,749abcd	0.99
ZC60	35,299	221i	2.48 h	217i	0.99
ZM160	43,430	176i	2.31 h	174i	0.99
ZM260	46,804	152i	1.25i	131i	0.99
GN60	39,463	306i	2.65 h	310i	0.99
WL60	39,979	1,040gh	4.37def	1,069gh	0.99
XJD60	50,367	1,119 g	4.28efg	1,129 g	0.99
SEM	1,450	128.09	0.27	127.04	–
*P*-value	0.533	<0.001	<0.001	<0.001	–

Values with different letters indicate significant differences between fresh materials and silages. ZC, Zhongcao No. 3; ZM1, Zhongmu No.1; ZM2, Zhongmu No.2; GN, Gongnong No.1; WL, WL168; XJD, xinjiangdaye. 0, 0 day of ensiling; 60, 60 days of ensiling.

**Figure 1:**
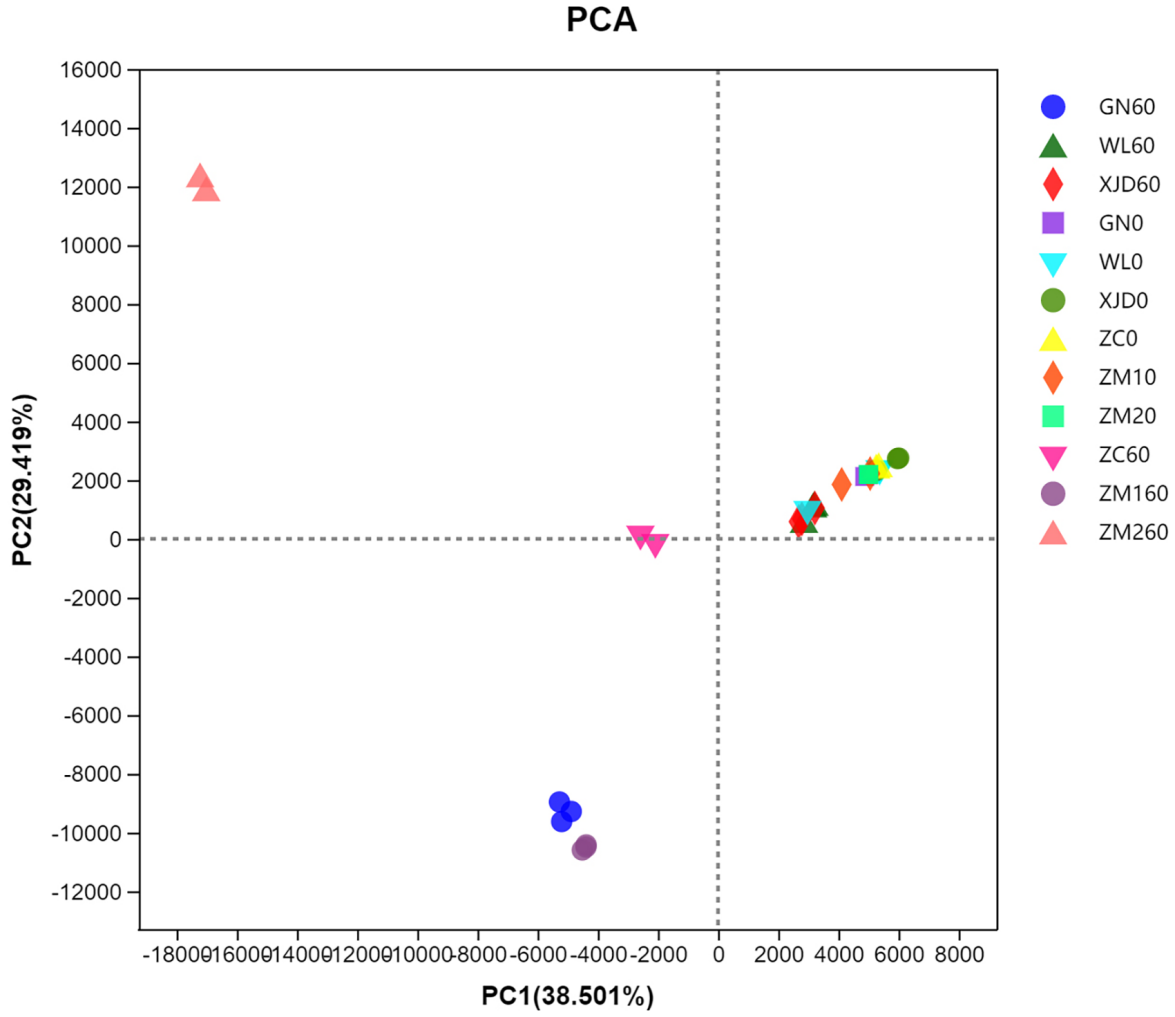
Principal component analysis (PCA) of the bacterial communities in silage and fresh materials. ZC, Zhongcao No. 3; ZM1, Zhongmu No. 1; ZM2, Zhongmu No. 2; GN, Gongnong No. 1; WL, WL168; XJD, xinjiangdaye; 0, 0 day of ensiling; 60, 60 days of ensiling.

PCA demonstrated the distribution of bacterial communities in each group of samples (Figure 1). The epiphytic bacterial communities of fresh alfalfa and alfalfa silage were clearly separated. The bacterial communities attached to fresh alfalfa showed clustering. After ensiling, samples GN and ZM1 were clustered, while WL and XJD were aggregated.

**Figure 2:**
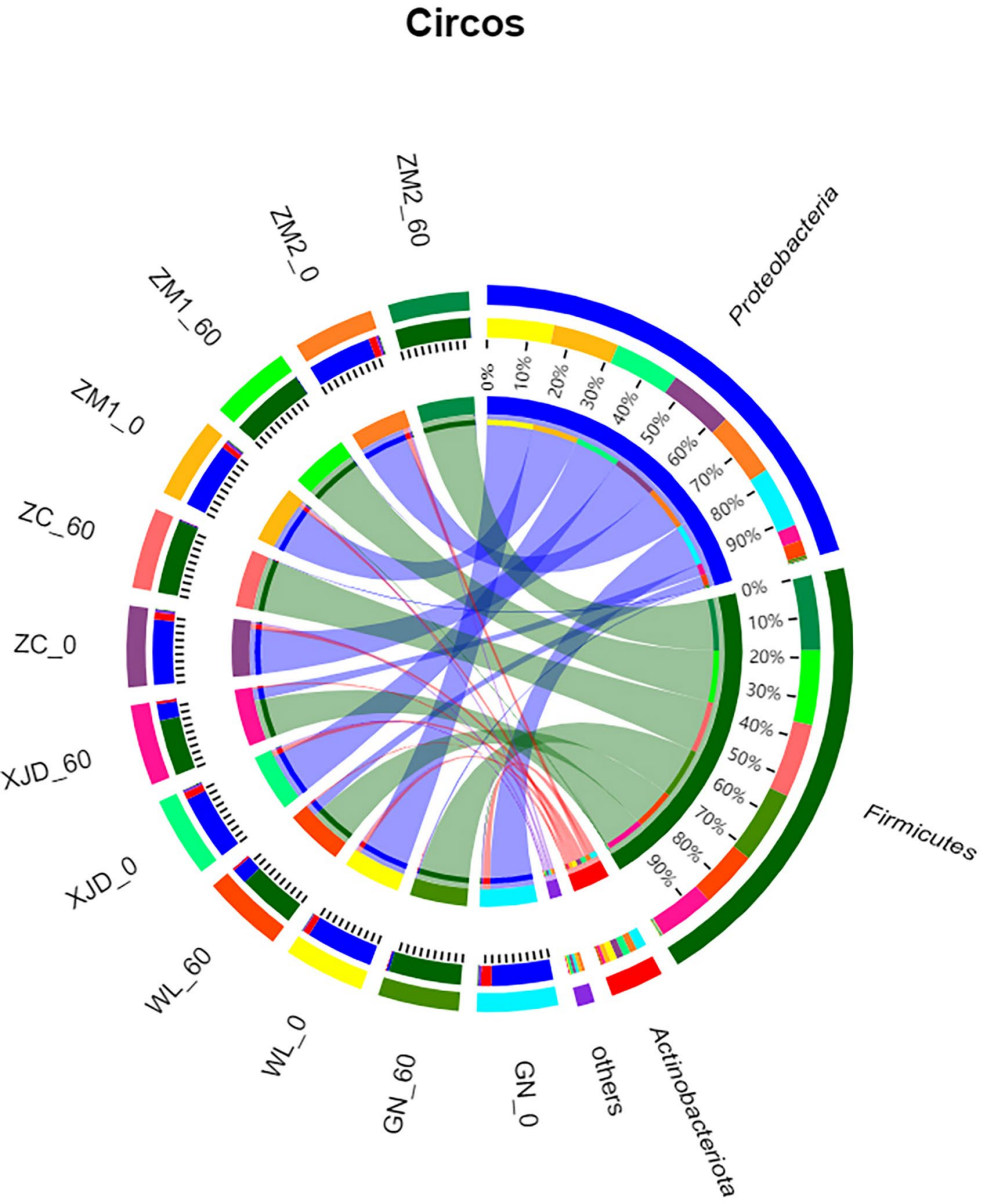
Circos plot showing the differences in bacterial species composition across samples at the phylum level. ZC, Zhongcao No. 3; ZM1, Zhongmu No. 1; ZM2, Zhongmu No. 2; GN, Gongnong No. 1; WL, WL168; XJD, xinjiangdaye; 0, 0 day of ensiling; 60, 60 days of ensiling.

The epiphytic bacterial community composition (phylum level) of fresh alfalfa and alfalfa silage is shown in Figure 2. Before ensiling, *Proteobacteria* and *Actinobacteriota* dominated at the phylum level for each alfalfa species. After ensiling, the dominant phylum of epiphytic bacteria changed to *Firmicutes*, and the abundance of *Proteobacteria* and *Actinobacteriota* decreased in all varieties of alfalfa.

**Figure 3:**
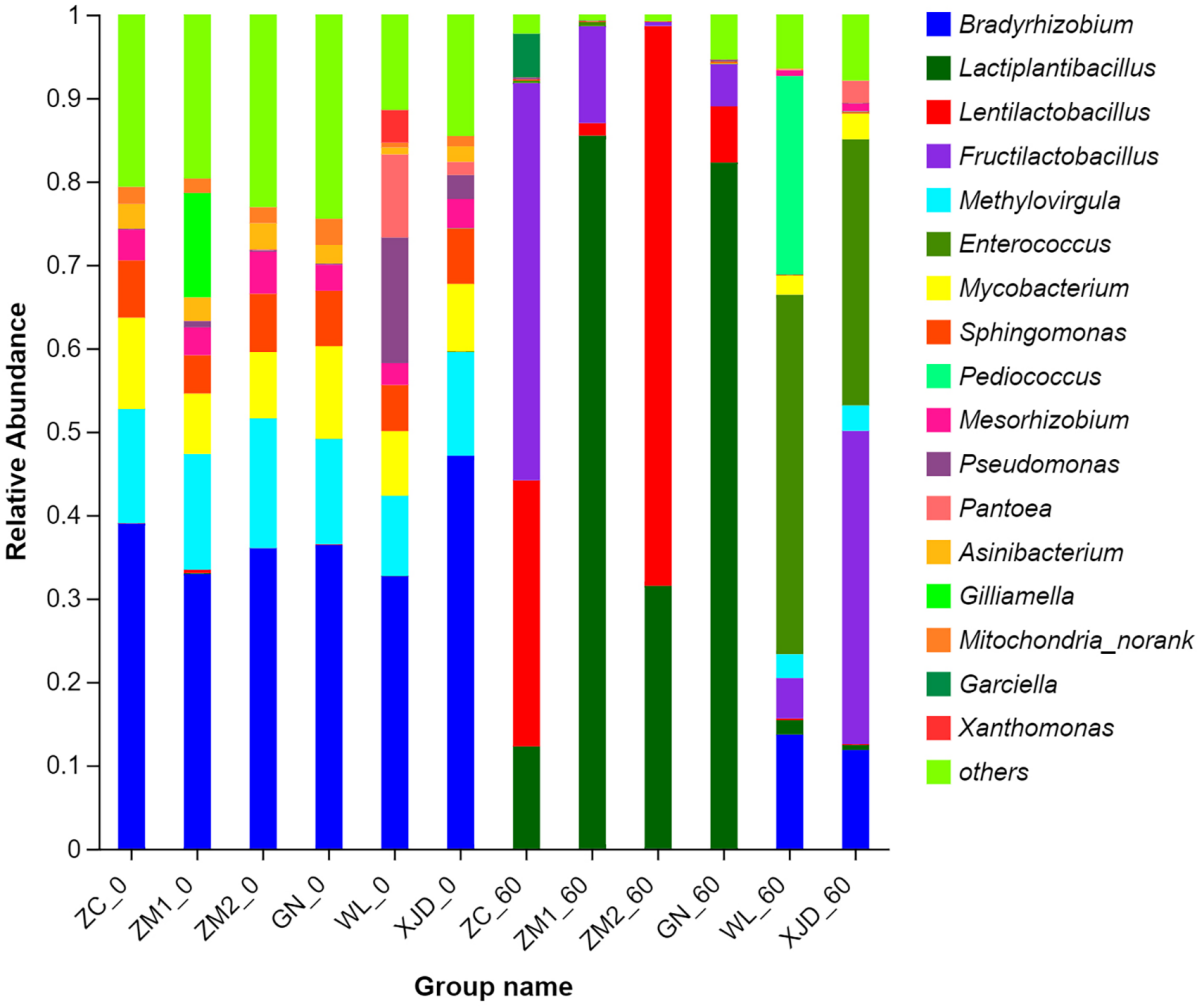
Bacterial community composition of fresh alfalfa and alfalfa silage at the genus level. ZC, Zhongcao No. 3; ZM1, Zhongmu No.1; ZM2, Zhongmu No. 2; GN, Gongnong No. 1; WL,WL168; XJD, xinjiangdaye; 0,0 day of ensiling; 60, 60 days of ensiling.

A stacked bar chart was generated to visualize the bacterial species composition at the silage genus level for each alfalfa species (Figure 3). Before ensiling, *Bradyrhizobium* was the dominant genus for ZC (38.98%), ZM1 (32.95%), ZM2 (36%), GN (36.42%), WL (32.68%) and XJD (47.09%). The abundance of *Bradyrhizobium* was significantly reduced in all alfalfa species after ensiling. The dominant genera in the ZC group were *Fructilactobacillus* (47.62%), *Lentilactobacillus* (31.92%), and *Lactiplantibacillus* (12.24%). The dominant genera in the ZM1 group were *Lactiplantibacillus* (85.48%) and *Fructilactobacillus* (11.60%). The dominant genera in the ZM2 group were *Lentilactobacillus* (67.10%) and *Lactiplantibacillus* (31.50%). The dominant genus in the GN group was *Lactiplantibacillus* (82.25%). Moreover, *Enterococcus* (43.11%), *Pediococcus* (23.83%) and *Bradyrhizobium* (13.67%) were the predominant genera in the WL group. The dominant genera in the XJD group were *Fructilactobacillus* (37.58%), *Enterococcus* (31.90%) and *Bradyrhizobium* (11.81%).

**Figure 4:**
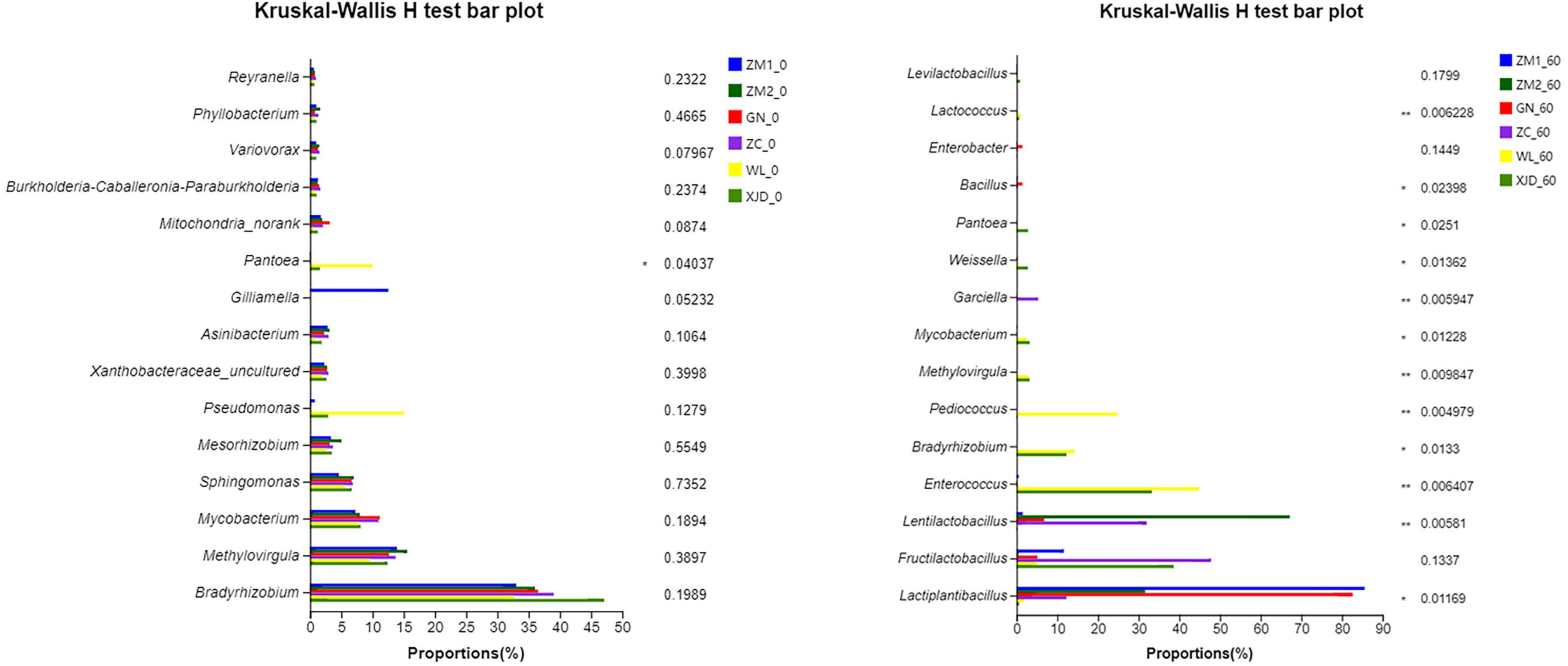
Comparison of microbial variations using the Kruskal-Wallis H test for alfalfa raw material and silage. ZC, Zhongcao No. 3; ZM1, Zhongmu No. 1; ZM2, Zhongmu No. 2; GN, Gongnong No. 1; WL, WL168; XJD, xinjiangdaye; 0, 0 day of ensiling; 60, 60 days of ensiling.

The variation in bacterial communities of each sample is depicted in Figure 4 (at the genus level). Before ensiling, except for *Pantoea*, there was no significant difference in the abundance of dominant epiphytic bacteria in various alfalfa varieties. The abundance of WL epiphytic *Pantoea* was significantly higher than the others. During the ensiling process, the abundance of alfalfa epiphytic dominant bacteria of all varieties decreased sharply. Accordingly, after ensiling, the dominant genera were *Lactiplantibacillus*, *Lentilactobacillus*, *Enterococcus*, *Bradyrhizobium*, and *Pediococcus*.

**Figure 5:**
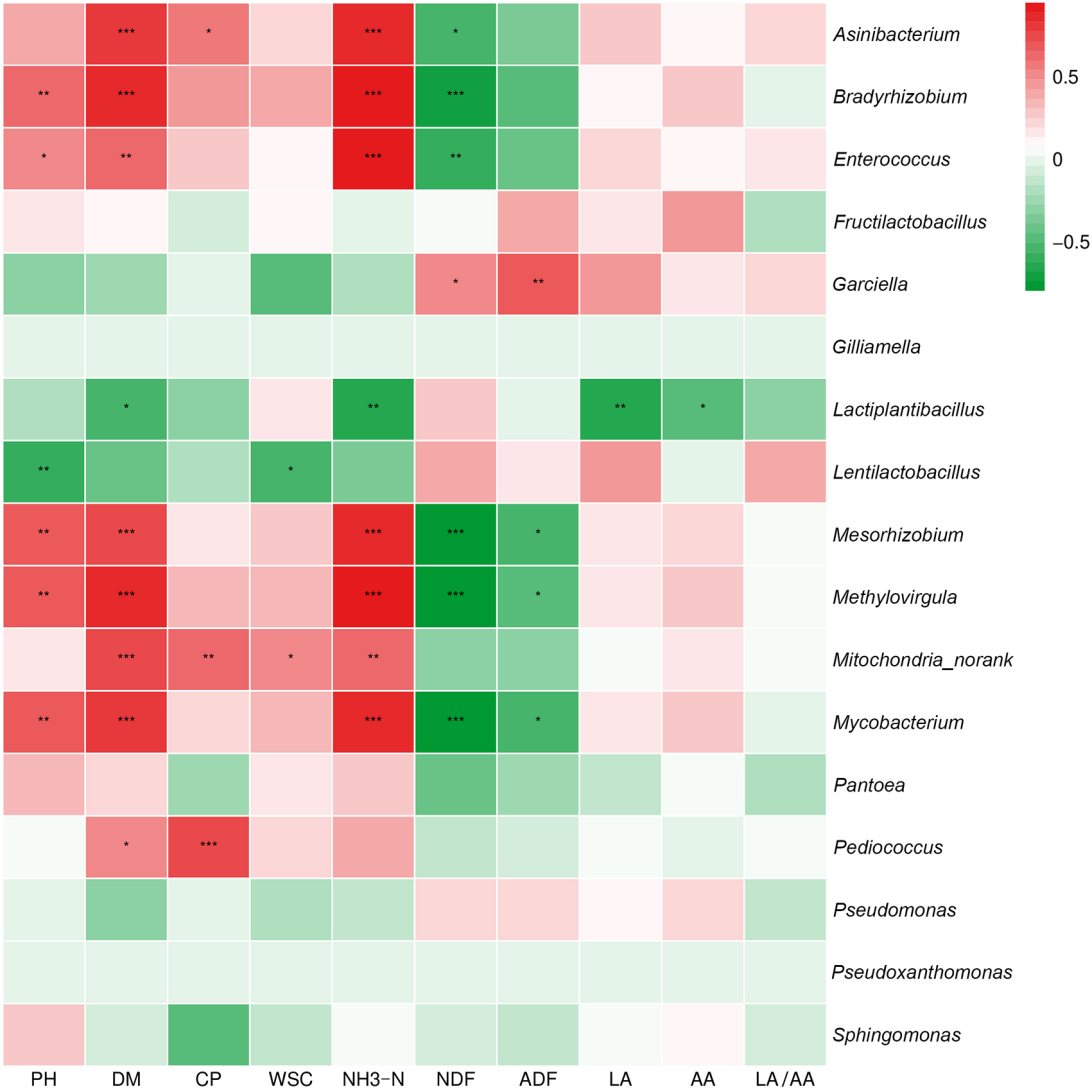
Correlation analysis of the high abundance of genus-level bacteria and fermentation quality in silage from different alfalfa varieties. *p < 0.05, **p < 0.01, and ***p < 0.001, respectively.

Pearson correlation analysis was conducted between bacteria and fermentation parameters at the genus level (Figure 5). The silage pH was positively correlated with the abundance of *Bradyrhizobium*, *Mesorhizobium*, *Methylovirgula* and *Mycobacterium* and negatively correlated with the abundance of *Lentilactobacillus* (*p* < 0.01). The silage DM was significantly correlated with the abundance of *Asinibacterium*, *Bradyrhizobium*, *Mesorhizobium*, *Methylovirgula*, *Mitochondria_norank* and *Mycobacterium* (*p* < 0.001) and negatively correlated with *Lactiplantibacillus* (*p* < 0.05). The silage CP was significantly positively correlated with the abundance of *Pediococcus* (*p* < 0.001), *Mitochondria_norank* (*p* < 0.01) and *Asinibacterium* (*p* < 0.05). The silage WSC showed a significant positive correlation with the abundance of *Mitochondria_norank* and a negative correlation with *Lentilactobacillus*. The silage NH_3_-N was highly significantly positively correlated with the abundance of *Asinibacterium*, *Bradyrhizobium*, *Enterococcus*, *Methylovirgula* and *Mycobacterium* (*p* < 0.001) and negatively correlated with the abundance of *Lactiplantibacillus* (*p* < 0.01). The silage NDF was negatively correlated (*p* < 0.001) with the abundance of *Bradyrhizobium*, *Mesorhizobium*, *Methylovirgula* and *Mycobacterium*. Moreover, the silage ADF was negatively correlated with the abundance of *Mesorhizobium*, *Methylovirgula* and *Mycobacterium* (*p* < 0.01). Finally, the silage LA and AA were negatively correlated with *Lactiplantibacillus* (*p* < 0.01, *p* < 0.05).

**Figure 6:**
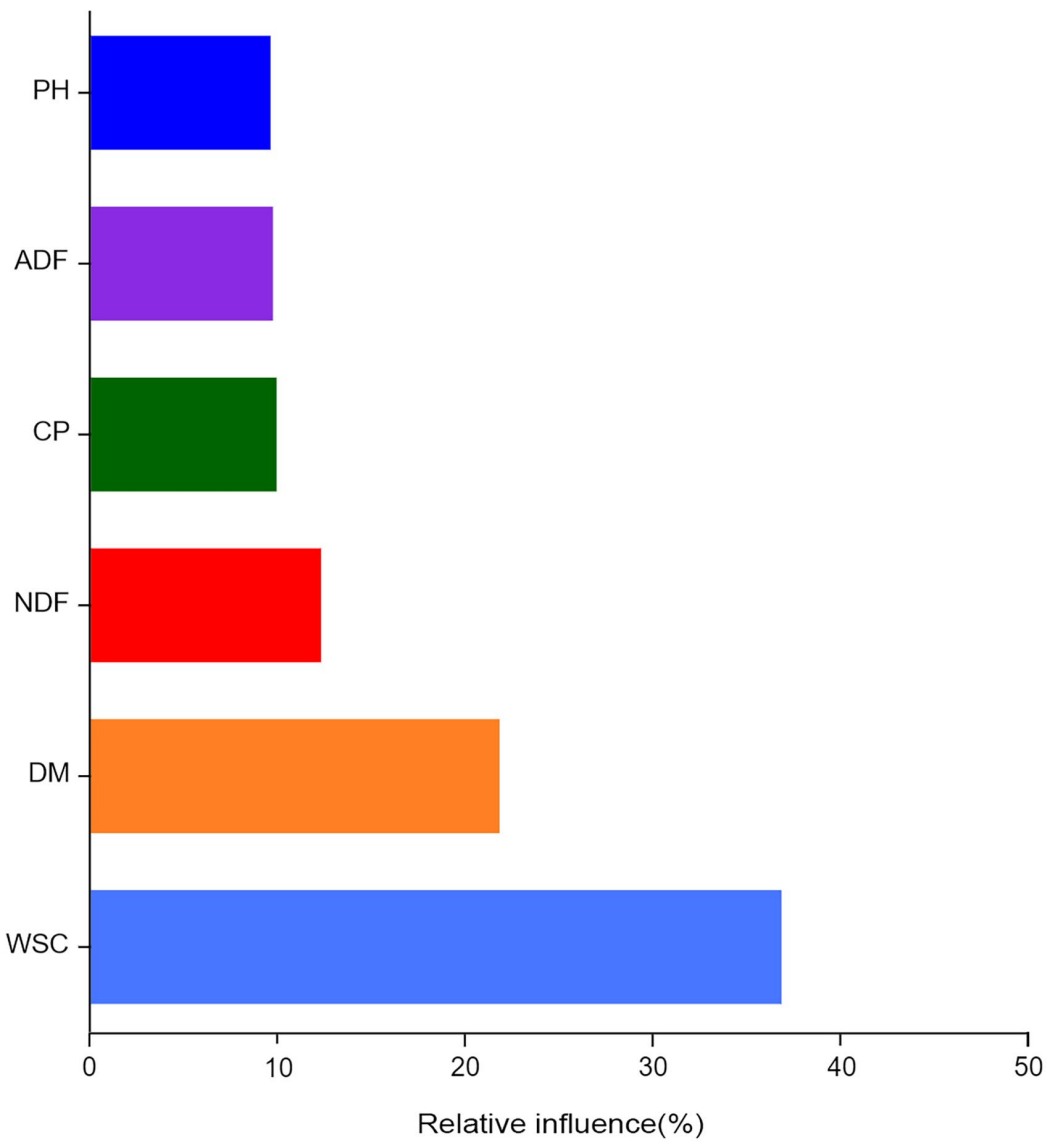
Aggregated boosted tree (ABT) analysis assessed the relative importance of fermentation quality and nutritional quality on bacterial community composition.

Aggregated boosted tree analysis was conducted to assess the relationship between the chemical composition of silage ingredients and the bacterial community of silage (Figure 6). The results showed that WSC was the most important factor affecting the bacterial community structure in alfalfa silage (36.79%), followed by DM (21.77%).

## Discussion

### Chemical composition of alfalfa before and after ensiling

It is well-established that the chemical composition of alfalfa can vary significantly depending on the variety, harvesting period and storage method (Shangli et al., 2017; Zheng et al., 2018). In this study, six alfalfa varieties were planted in the same experimental field and harvested during the same period. Our findings suggest that the alfalfa variety is the main factor accounting for the heterogeneity in the chemical composition of alfalfa.

It has been shown that the fermentation of *Clostridium* can be inhibited when the DM content exceeds 300 g/kg FM (Leibensperger and Pitt, 1987). In alfalfa silage, higher DM content can inhibit the growth of AA and BA producing microorganisms (Whiter and Kung Jr., 2001). In this study, the DM content of the six varieties of fresh alfalfa ranged from 463 g/kg FM to 513 g/kg FM, which met the optimal criteria for alfalfa silage fermentation. After ensiling, there was no significant change in DM content in all groups, except for the WL and XJD groups, where DM content was elevated. This finding indicates that the silage fermentation process greatly preserved the nutritional quality of alfalfa. Accordingly, the CP content is an important indicator for assessing the nutritional value of feed, and the higher the CP content, the higher the nutritional value of the feed. In this study, the CP content of each alfalfa variety before ensiling ranged from 166 g/kg DM to 222 g/kg DM. There was no significant difference in the CP content of the three groups ZC, ZM2, GN and WL after ensiling, indicating that the silage process preserved the nutritional value of these four alfalfa species to a large extent. The CP content of ZM1 was significantly increased after ensiling (*p* < 0.05), which may be attributed to significant protein degradation into soluble proteins under the action of proteolytic enzymes during the ensiling process, which led to decreased non-degradable protein content and significantly increased content of soluble crude protein and non-protein nitrogen (Papadopoulos and McKersie, 1982; Rooke and Armstrong, 1989). However, the CP content of the XJD group was reduced, which may be due to plant respiration and protein hydrolysis in the pre-silage period (Li et al., 2020). Initial WSC content ranged from 60 to 70 g/kg DM was deemed as the optimal requirement to achieve well-preserved fermentation (Zheng et al., 2018). During silage fermentation, LAB can use WSC on crops to produce organic acids and reduce the pH value of silage (Yang et al., 2020). Consistent with the literature, we found that the WSC content was reduced after ensiling (Bernardes et al., 2018; Ogunade et al., 2018). Interestingly, both WL and XJD with higher WSC content produced less LA than ZC and ZM2. This might be inhibited by the higher DM. Previous studies have shown that when the DM content exceeds 500 g/kg FM, all microorganisms, including LAB, are inhibited (Muck, 1990).

### Fermentation quality of silage

During the silage fermentation process, the pH value, NH_3_-N, and organic acids are important indicators for assessing silage fermentation quality. Generally, the pH value of legume silage ranges from 4.3 to 5.0 (Kung et al., 2018). In this study, the pH values of the six varieties of alfalfa silage ranged from 4.5 to 5.3, slightly higher than previously reported. The variation in PH values could be due to the heterogeneous chemical composition of different alfalfa varieties or differences in silage microbial communities, consistent with previous reports by Tremblay et al. (2001). NH_3_-N reflects the degradation of proteins in silage, a process mainly mediated by plant and microbial proteases (Ohshima and McDonald, 1978). Interestingly, the type and activity of proteases may also vary during the ensiling process, depending on the alfalfa species and microbial community. There is an increasing consensus that for forages of good silage quality, NH_3_-N should be less than 100 g/kg TN (Yuan et al., 2012; Kung et al., 2018). In the present study, the relatively low NH_3_-N/TN content of the ZC, ZM1, ZM2, and GN groups indicated that protein loss after ensiling was relatively low. However, the content of NH_3_-N/TN in both WL and XJD groups was higher than 100 g/kg TN, indicating that protein loss was more significant after ensiling in these two varieties of alfalfa. In general, the higher protein loss may be due to the activity of *Clostridium*, which in turn produces higher amounts of BA (Pahlow et al., 2003). However, in the present study, BA was not detected in the silages in both WL and XJD groups. This suggested the involvement of other bacteria in protein degradation, such as *Mycobacterium*, *Enterococcus* and *Weissella* (Lu et al., 2021; Bao et al., 2022).

Organic acids are products of lactic acid bacteria metabolism during silage fermentation that can lower the pH in the feed, thereby inhibiting the activity of microorganisms associated with protein hydrolysis. Among them, LA was the largest contributor to lowering pH and usually accounts for most silage (Fan et al., 2021). Therefore, the high LA content and low pH of the ZC group were attributed to the rapid production of LA by LAB and the reduction of pH in a short period. In general, AA is the second highest acid in silage; studies have shown that AA has strong antifungal properties, and an appropriate amount of AA content can increase the aerobic stability of silage (Kung et al., 2000; Bai et al., 2020). In the present study, there were significant differences in LA and AA concentrations of alfalfa forages among varieties after ensiling, which may be due to differences in the chemical properties of different alfalfa and microbial communities during the fermentation process. Current evidence suggests that propionic acid bacteria can produce PA in large quantities during silage fermentation, and their role is mainly to improve the aerobic stability of silage. However, high concentrations of PA and BA are usually found in *Clostridium* fermented silage and indicate poor fermentation (Kung et al., 2018). In this study, BA and PA were not detected in all groups of silages, which may be due to the killing of *Clostridium* spores during the wilting process in fresh alfalfa (Zheng et al., 2018).

### Bacterial community of silage

In this study, the Good’s coverage index of all samples reached approximately 1, indicating that most bacteria were detected. Alpha diversity analysis was conducted to assess the microbial abundance and species richness contained in the sample. The ACE and Chao1 index reflect the richness of microbial species, and the Shannon index reflects the richness and evenness of species (Fu et al., 2022). Consistent with the literature (Zhang et al., 2022), we found that the microbial community composition of alfalfa epiphytes grown in the same season and region was less influenced by species. The abundance and species richness of the six alfalfa epiphytic bacterial communities were similar. The PCA plots further demonstrated that the six alfalfa epiphytic microbial communities were similar before ensiling. Similar to the work of Blajman et al. (2020) and Lu et al. (2021), the abundance and diversity of the bacterial community decreased after ensiling. During the ensiling process, aerobic bacteria and partly aerobic bacteria rapidly consume oxygen in the environment and create an anaerobic environment, causing inhibited metabolic activities of many microorganisms in the raw material and eventually replaced by anaerobic or facultative anaerobic bacteria such as lactic acid bacteria (Muck et al., 2018). In the present study, the abundance of *Bradyrhizobium*, *Methylovirgula*, *Mycobacterium* and *Sphingomonas* declined or even disappeared, leading to a decrease in bacterial diversity. Eventually, *Lactiplantibacillus*, *Fructilactobacillus* and *Lentilactobacillus* became the dominant genera for silage.

In the present study, the dominant genera before ensiling were *Proteobacteria* and *Actinobacteriota* and shifted to *Firmicutes* after ensiling. Nazar et al. (2020) similarly reported a shift in the bacterial community from the *Proteobacteria* to the *Firmicutes* and advocated that anaerobic and acidic environments contribute to the growth of *Firmicutes*. Microbial community structure and diversity during ensiling are important factors affecting fermentation (Figure 3). *Bradyrhizobium* and *Mycobacterium* were the main epiphytic genera of fresh alfalfa before ensiling. *Bradyrhizobium* is a common genus of rhizobia in legumes, named for its slow growth and symbiotic relationship during alfalfa growth (Fred et al., 1932). The gram-positive bacterium *Mycobacterium* is a genus in the family *Mycobacterium*. Some *Mycobacterium* species, such as *Mycobacterium Bovis*, are pathogenic and can reportedly cause tuberculosis in humans and animals (Grooms et al., 2019).

After ensiling, the main genera in alfalfa silage were *Lactiplantibacillus*, *Lentilactobacillus*, *Fructilactobacillus*, *Pediococcus* and *Enterococcus* (Figure 3). In this respect, it has been shown that *Fructilactobacillus* can use fructose to produce organic acids such as LA and AA, often present in yeast cultures or sourdough (Martau et al., 2021), but were rarely seen during silage fermentation. In the present study, the ZC and XJD groups had a higher abundance of *Fructilactobacillus*, attributed to the fermentation process promoting the breakdown of fructose in both alfalfa species, which led to an increased abundance of *Fructilactobacillus*. Studies have shown that *Lactiplantibacillus* and *Lentilactobacillus* were ideal functional bacteria after ensiling and can be used to improve silage quality (Fu et al., 2022; Li et al., 2022). During the fermentation process, *Lactiplantibacillus* can inhibit the growth of *Clostridium*, thereby reducing the content of ammonium nitrogen in the feed, resulting in a higher fermentation quality of the silage (Du et al., 2022). Consistently, in the present study, *Lactiplantibacillus* showed a negative correlation with NH_3_-N/TN. It is generally believed that during the pre-ensiling stage, *Enterococcus* and *Pediococcus* can rapidly produce LA and promote the formation of an anaerobic environment to facilitate LAB growth. However, it should be borne in mind that *Enterococcus* and *Pediococcus* are less acid-tolerant and are thus replaced by the more acid-tolerant *Lactiplantibacillus* and *Fructilactobacillus* during the late ensiling stage (Parvin et al., 2010), accounting for the positive correlation between pH and *Enterococcus* (*p* < 0.05). Besides, in this study, the LA/AA values of all alfalfa forages except ZM2 were less than 2, which indicated that the remaining five alfalfa silages were mainly based on acetic acid fermentation. Anisotropic fermenting LAB can convert LA to AA, resulting in lower LA/AA values (Nair et al., 2020).

In the present study, the chemical composition of the six alfalfa feedstocks differed significantly by species, but the epiphytic bacterial communities were similar at the genus level. Correlation analysis showed that the differences in the chemical composition of different alfalfa varieties significantly affected the fermentation quality and bacterial community composition of silage; WSC and DM were the main drivers of bacterial community changes during alfalfa during alfalfa ensiling. Higher WSC could provide more carbon sources for microbial activities, thus favoring the sugar-fermenting microorganisms (Hisham et al., 2022). Silage materials with lower DM content have more water than those with higher DM content. Microorganisms can harness this water better for metabolism, which leads to a faster decrease in silage pH for low DM content than for high DM content (Kung et al., 2018). Thus WSC and DM can significantly influence the changes in the bacterial flora during alfalfa ensiling.

## Conclusion

This study investigated the chemical composition, fermentation quality and microbial composition of six alfalfa species harvested at the same time, region and fertility period. After ensiling, ZM2 exhibited a lower pH and NH_3_-N content and a higher LA content compared to other varieties of alfalfa silage. Moreover, beneficial bacteria such as *Lentilactobacillus* and *Lactiplantibacillus* were predominant, accounting for the high fermentation quality of ZM2. The chemical composition of different alfalfa varieties before ensiling was different, while the epiphytic microbial communities were similar. After ensiling, there were significant differences in the fermentation quality and microbial community composition of different alfalfa varieties. In a nutshell, the chemical composition of silage materials determined the bacterial community composition of silage, among which WSC and DM content were important determining factors.

## Data availability statement

The data presented in the study are deposited in the NCBI Sequence Read Archive (SRA) repository, accession number SRA412564.

## Author contributions

JL: methodology, visualization, and data curation. GL: experimental design and implementation of some experiments. LinS: conceptualization, acquisition, reviewing, and editing. SW, XM, and LicS: interpret the data and edit the language. LY and LX: conceive the study and review the manuscript. All authors who contributed to the manuscript have read and approved the final version.

## Funding

This research was received financial support from the Natural Science Foundation of Inner Mongolia (No. 2020ZD08) and Science and Technology project of the Inner Mongolia Autonomous Region (No. 2021GG0157).

## Conflict of interest

The authors declare that the research was conducted in the absence of any commercial or financial relationships that could be construed as a potential conflict of interest.

## Publisher’s note

All claims expressed in this article are solely those of the authors and do not necessarily represent those of their affiliated organizations, or those of the publisher, the editors and the reviewers. Any product that may be evaluated in this article, or claim that may be made by its manufacturer, is not guaranteed or endorsed by the publisher.
